# *catena*-Poly[zinc(II)-bis­[μ-2-(2,4-dichloro­phen­oxy)acetato]]

**DOI:** 10.1107/S1600536810015977

**Published:** 2010-05-08

**Authors:** Shi-Zhu Liu

**Affiliations:** a School of Chemistry and Environment, South China Normal University, Guangzhou 510631, People’s Republic of China

## Abstract

The title polymeric compound, [Zn(C_8_H_5_Cl_2_O_3_)_2_]_*n*_, was prepared by reaction of zinc(II) chloride with 2,4-dichloro­phenoxy­acetic acid and sodium hydroxide under hydro­thermal conditions. The Zn^II^ atom is coordinated in a distorted tetra­hedral environment by four O atoms from four 2,4-dichloro­phenoxy­acetate ligands. Each ligand bridges two Zn^II^ atoms, forming a polymeric chain along the *a* axis. Adjacent chains are connected *via* C—H⋯Cl hydrogen bonds.

## Related literature

For metal-organic coordination polymers, see: Qin *et al.* (2009 ▶); Huang *et al.* (2008 ▶); Reineke *et al.* (1999 ▶); Xiong *et al.* (2002 ▶).
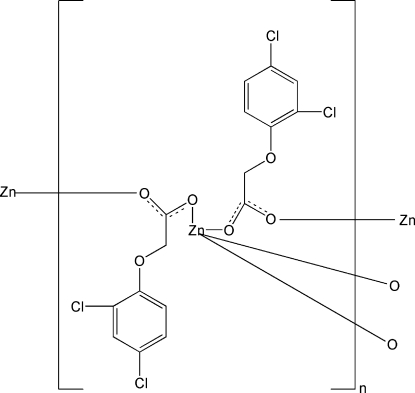


## Experimental

### 

#### Crystal data

[Zn(C_8_H_5_Cl_2_O_3_)_2_]
*M* *_r_* = 505.43
Triclinic, 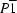

*a* = 4.7322 (10) Å
*b* = 10.459 (2) Å
*c* = 18.979 (4) Å
α = 79.340 (2)°
β = 89.838 (2)°
γ = 82.847 (3)°
*V* = 915.8 (3) Å^3^
*Z* = 2
Mo *K*α radiation
μ = 1.96 mm^−1^
*T* = 296 K
0.56 × 0.21 × 0.16 mm

#### Data collection

Bruker SMART APEXII CCD diffractometer
Absorption correction: multi-scan (*SADABS*; Sheldrick, 1996 ▶) *T*_min_ = 0.617, *T*_max_ = 0.731
4737 measured reflections
3255 independent reflections
2849 reflections with *I* > 2σ(*I*)
*R*_int_ = 0.021

#### Refinement

*R*[*F* ^2^ > 2σ(*F* ^2^)] = 0.030
*wR*(*F* ^2^) = 0.091
*S* = 0.94
3255 reflections
244 parameters
H-atom parameters constrained
Δρ_max_ = 0.34 e Å^−3^
Δρ_min_ = −0.39 e Å^−3^

### 

Data collection: *APEX2* (Bruker, 2004 ▶); cell refinement: *SAINT* (Bruker, 2004 ▶); data reduction: *SAINT*; program(s) used to solve structure: *SHELXS97* (Sheldrick, 2008 ▶); program(s) used to refine structure: *SHELXL97* (Sheldrick, 2008 ▶); molecular graphics: *SHELXTL* (Sheldrick, 2008 ▶); software used to prepare material for publication: *SHELXTL*.

## Supplementary Material

Crystal structure: contains datablocks I, global. DOI: 10.1107/S1600536810015977/ci5084sup1.cif

Structure factors: contains datablocks I. DOI: 10.1107/S1600536810015977/ci5084Isup2.hkl

Additional supplementary materials:  crystallographic information; 3D view; checkCIF report

## Figures and Tables

**Table 1:** Hydrogen-bond geometry (Å, °)

*D*—H⋯*A*	*D*—H	H⋯*A*	*D*⋯*A*	*D*—H⋯*A*
C2—H2*B*⋯Cl2^i^	0.97	2.73	3.610 (3)	151

Symmetry code: (i) 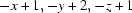.

**Fig. 1.**
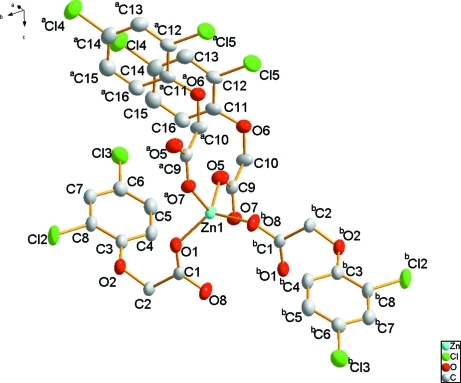
Part of a polymeric chain in the title compound. Displacement ellipsoids are drawn at the 50% probability level. Symmetry codes: (a) 1+x, y, z; (b) 1-x, 1-y, 1-z.

**Fig. 2.**
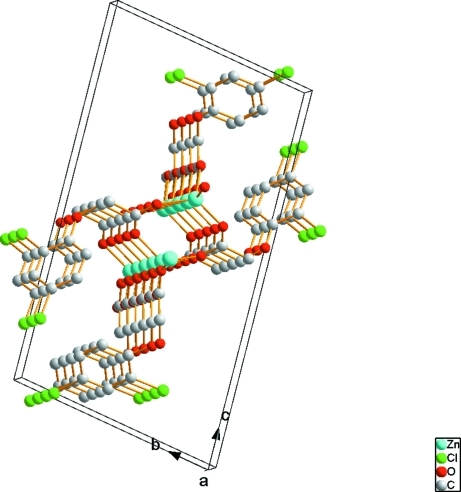
Part of the crystal packing, showing a polymeric chain viewed along the a axis. H atoms have been omitted for clarity.

## supplementary crystallographic information

### Experimental

A mixture of ZnCl_2_ (0.068 g, 0.5 mmol), H_2_O (6 ml), 2,4-dichlorophenoxyacetic acid (0.22 g, 1 mmol) was stirred vigorously for 10 min and then sodium hydroxide solution (1.0 mol/L) was added to adjust the pH value to 7.0. The mixture was then sealed in a 25 ml Teflon-lined stainless-steel autoclave. The autoclave was heated at 413 K for 3 d and then slowly cooled to room temperature at 6 K/h. The product was collected by filtration, washed with water and air-dried. Colourless needle-shaped crystals were obtained in ca. 42.3% yield based on Zn.

### Refinement

H atoms were positioned geometrically [C–H = 0.93–0.97 Å] and refined using a riding model, with *U*_iso_(H) = 1.2*U*_eq_(C).

### Crystal data

**Table:**

[Zn(C_8_H_5_Cl_2_O_3_)_2_]	*Z* = 2
*M**_r_* = 505.43	*F*(000) = 504
Triclinic, *P*1	*D*_x_ = 1.833 Mg m^−^^3^
Hall symbol: -P 1	Mo *K*α radiation, λ = 0.71073 Å
*a* = 4.7322 (10) Å	Cell parameters from 2642 reflections
*b* = 10.459 (2) Å	θ = 2.5–27.7°
*c* = 18.979 (4) Å	µ = 1.96 mm^−^^1^
α = 79.340 (2)°	*T* = 296 K
β = 89.838 (2)°	Needle, colourless
γ = 82.847 (3)°	0.56 × 0.21 × 0.16 mm
*V* = 915.8 (3) Å^3^	

### Data collection

**Table:**

Bruker SMART APEXII CCD diffractometer	3255 independent reflections
Radiation source: fine-focus sealed tube	2849 reflections with *I* > 2σ(*I*)
graphite	*R*_int_ = 0.021
ω scans	θ_max_ = 25.2°, θ_min_ = 2.0°
Absorption correction: multi-scan (*SADABS*; Sheldrick, 1996)	*h* = −5→5
*T*_min_ = 0.617, *T*_max_ = 0.731	*k* = −12→8
4737 measured reflections	*l* = −22→21

### Refinement

**Table:**

Refinement on *F*^2^	Primary atom site location: structure-invariant direct methods
Least-squares matrix: full	Secondary atom site location: difference Fourier map
*R*[*F*^2^ > 2σ(*F*^2^)] = 0.030	Hydrogen site location: inferred from neighbouring sites
*wR*(*F*^2^) = 0.091	H-atom parameters constrained
*S* = 0.94	*w* = 1/[σ^2^(*F*_o_^2^) + (0.0663*P*)^2^] where *P* = (*F*_o_^2^ + 2*F*_c_^2^)/3
3255 reflections	(Δ/σ)_max_ = 0.024
244 parameters	Δρ_max_ = 0.34 e Å^−^^3^
0 restraints	Δρ_min_ = −0.39 e Å^−^^3^

### Special details

**Table:**

Geometry. All esds (except the esd in the dihedral angle between two l.s. planes) are estimated using the full covariance matrix. The cell esds are taken into account individually in the estimation of esds in distances, angles and torsion angles; correlations between esds in cell parameters are only used when they are defined by crystal symmetry. An approximate (isotropic) treatment of cell esds is used for estimating esds involving l.s. planes.
Refinement. Refinement of *F*^2^ against ALL reflections. The weighted *R*-factor wR and goodness of fit *S* are based on *F*^2^, conventional *R*-factors *R* are based on *F*, with *F* set to zero for negative *F*^2^. The threshold expression of *F*^2^ > σ(*F*^2^) is used only for calculating *R*-factors(gt) etc. and is not relevant to the choice of reflections for refinement. *R*-factors based on *F*^2^ are statistically about twice as large as those based on *F*, and *R*- factors based on ALL data will be even larger.

### Fractional atomic coordinates and isotropic or equivalent isotropic displacement parameters (Å^2^)

**Table:**

	*x*	*y*	*z*	*U*_iso_*/*U*_eq_	
Zn1	0.66929 (6)	0.53465 (3)	0.409892 (14)	0.02622 (12)	
Cl2	0.46808 (18)	1.18539 (7)	0.33205 (4)	0.0500 (2)	
Cl3	−0.18417 (19)	1.02366 (8)	0.14274 (4)	0.0538 (2)	
O1	0.5602 (4)	0.69621 (17)	0.44411 (10)	0.0354 (4)	
C2	0.2487 (7)	0.8626 (2)	0.48725 (14)	0.0368 (6)	
H2A	0.0425	0.8686	0.4858	0.044*	
H2B	0.3034	0.8854	0.5321	0.044*	
C1	0.3707 (5)	0.7213 (2)	0.48733 (13)	0.0283 (5)	
O2	0.3338 (4)	0.95654 (17)	0.43021 (10)	0.0381 (4)	
C8	0.2572 (6)	1.0724 (2)	0.31164 (15)	0.0344 (6)	
C3	0.2031 (6)	0.9673 (2)	0.36461 (14)	0.0336 (6)	
C7	0.1426 (6)	1.0904 (3)	0.24305 (15)	0.0374 (6)	
H7	0.1846	1.1593	0.2077	0.045*	
C4	0.0218 (6)	0.8825 (3)	0.34807 (15)	0.0370 (6)	
H4	−0.0198	0.8129	0.3830	0.044*	
C6	−0.0369 (6)	1.0027 (3)	0.22834 (15)	0.0381 (6)	
C5	−0.0982 (6)	0.9003 (3)	0.27995 (16)	0.0409 (7)	
H5	−0.2199	0.8429	0.2693	0.049*	
O5	0.4663 (4)	0.5250 (2)	0.32238 (10)	0.0379 (4)	
C9	0.2002 (5)	0.5268 (2)	0.32394 (13)	0.0285 (5)	
C10	0.0513 (6)	0.4985 (3)	0.25952 (14)	0.0365 (6)	
H10A	−0.0637	0.4283	0.2752	0.044*	
H10B	−0.0762	0.5759	0.2383	0.044*	
O6	0.2391 (4)	0.46218 (19)	0.20660 (10)	0.0394 (5)	
C11	0.3544 (6)	0.5571 (3)	0.16035 (14)	0.0335 (6)	
C12	0.5536 (6)	0.5123 (3)	0.11352 (14)	0.0371 (6)	
C14	0.6058 (7)	0.7315 (3)	0.05874 (16)	0.0440 (7)	
C15	0.4104 (7)	0.7786 (3)	0.10480 (18)	0.0481 (7)	
H15	0.3623	0.8684	0.1018	0.058*	
C13	0.6794 (7)	0.5995 (3)	0.06313 (15)	0.0457 (7)	
H13	0.8135	0.5685	0.0323	0.055*	
C16	0.2865 (7)	0.6908 (3)	0.15540 (17)	0.0452 (7)	
H16	0.1552	0.7224	0.1866	0.054*	
Cl5	0.6472 (2)	0.34585 (8)	0.11910 (5)	0.0616 (3)	
Cl4	0.7620 (2)	0.84005 (10)	−0.00545 (5)	0.0660 (3)	
O7	0.0604 (4)	0.54895 (18)	0.37772 (9)	0.0331 (4)	
O8	0.2660 (4)	0.64018 (18)	0.53311 (11)	0.0449 (5)	

### Atomic displacement parameters (Å^2^)

**Table:**

	*U*^11^	*U*^22^	*U*^33^	*U*^12^	*U*^13^	*U*^23^
Zn1	0.02447 (18)	0.02421 (18)	0.02907 (18)	−0.00534 (12)	0.00625 (12)	−0.00117 (12)
Cl2	0.0636 (5)	0.0330 (4)	0.0527 (5)	−0.0225 (4)	−0.0055 (4)	0.0049 (3)
Cl3	0.0667 (5)	0.0505 (5)	0.0438 (4)	−0.0079 (4)	−0.0080 (4)	−0.0074 (3)
O1	0.0368 (10)	0.0253 (9)	0.0450 (11)	−0.0078 (8)	0.0112 (9)	−0.0070 (8)
C2	0.0508 (17)	0.0239 (13)	0.0339 (14)	−0.0042 (12)	0.0067 (12)	−0.0011 (11)
C1	0.0325 (14)	0.0239 (12)	0.0274 (13)	−0.0053 (10)	−0.0012 (10)	−0.0002 (10)
O2	0.0522 (12)	0.0239 (9)	0.0361 (10)	−0.0091 (8)	0.0002 (9)	0.0025 (8)
C8	0.0348 (15)	0.0244 (13)	0.0427 (15)	−0.0077 (11)	0.0036 (12)	−0.0007 (11)
C3	0.0385 (15)	0.0225 (13)	0.0383 (14)	−0.0022 (11)	0.0054 (12)	−0.0030 (11)
C7	0.0443 (17)	0.0260 (13)	0.0390 (15)	−0.0049 (12)	0.0062 (12)	0.0018 (11)
C4	0.0425 (16)	0.0223 (13)	0.0433 (15)	−0.0063 (11)	0.0061 (12)	0.0026 (11)
C6	0.0412 (16)	0.0328 (14)	0.0388 (15)	0.0008 (12)	0.0007 (12)	−0.0063 (12)
C5	0.0425 (16)	0.0291 (14)	0.0518 (17)	−0.0075 (12)	0.0023 (13)	−0.0070 (12)
O5	0.0200 (9)	0.0564 (12)	0.0392 (10)	−0.0081 (8)	0.0039 (8)	−0.0116 (9)
C9	0.0257 (13)	0.0277 (13)	0.0306 (13)	−0.0048 (10)	0.0023 (10)	−0.0011 (10)
C10	0.0277 (14)	0.0499 (17)	0.0334 (14)	−0.0084 (12)	0.0027 (11)	−0.0091 (12)
O6	0.0429 (11)	0.0428 (11)	0.0352 (10)	−0.0103 (9)	0.0099 (8)	−0.0117 (9)
C11	0.0323 (14)	0.0419 (15)	0.0277 (13)	−0.0034 (12)	−0.0028 (11)	−0.0107 (11)
C12	0.0402 (16)	0.0382 (15)	0.0320 (14)	0.0038 (12)	0.0012 (11)	−0.0105 (12)
C14	0.0419 (17)	0.0481 (18)	0.0391 (16)	−0.0075 (14)	0.0001 (13)	0.0007 (13)
C15	0.0489 (18)	0.0373 (16)	0.0570 (19)	−0.0018 (14)	−0.0011 (15)	−0.0080 (14)
C13	0.0485 (18)	0.0522 (19)	0.0352 (15)	0.0003 (15)	0.0085 (13)	−0.0096 (14)
C16	0.0443 (17)	0.0421 (17)	0.0506 (17)	−0.0006 (14)	0.0137 (14)	−0.0152 (14)
Cl5	0.0829 (7)	0.0394 (4)	0.0605 (5)	0.0066 (4)	0.0191 (5)	−0.0135 (4)
Cl4	0.0775 (6)	0.0650 (6)	0.0514 (5)	−0.0220 (5)	0.0086 (4)	0.0081 (4)
O7	0.0256 (9)	0.0427 (11)	0.0335 (10)	−0.0108 (8)	0.0070 (8)	−0.0095 (8)
O8	0.0527 (13)	0.0266 (10)	0.0495 (12)	−0.0031 (9)	0.0156 (10)	0.0071 (9)

### Geometric parameters (Å, °)

**Table:**

Zn1—O1	1.9315 (18)	C5—H5	0.93
Zn1—O8^i^	1.9335 (18)	O5—C9	1.258 (3)
Zn1—O5	1.9458 (18)	C9—O7	1.256 (3)
Zn1—O7^ii^	1.9622 (18)	C9—C10	1.507 (3)
Cl2—C8	1.735 (3)	C10—O6	1.412 (3)
Cl3—C6	1.735 (3)	C10—H10A	0.97
O1—C1	1.250 (3)	C10—H10B	0.97
C2—O2	1.415 (3)	O6—C11	1.365 (3)
C2—C1	1.518 (3)	C11—C16	1.382 (4)
C2—H2A	0.97	C11—C12	1.392 (4)
C2—H2B	0.97	C12—C13	1.382 (4)
C1—O8	1.245 (3)	C12—Cl5	1.726 (3)
O2—C3	1.372 (3)	C14—C13	1.369 (4)
C8—C3	1.395 (4)	C14—C15	1.380 (5)
C8—C7	1.383 (4)	C14—Cl4	1.735 (3)
C3—C4	1.383 (4)	C15—C16	1.383 (4)
C7—C6	1.389 (4)	C15—H15	0.93
C7—H7	0.93	C13—H13	0.93
C4—C5	1.385 (4)	C16—H16	0.93
C4—H4	0.93	O7—Zn1^iii^	1.9622 (17)
C6—C5	1.371 (4)	O8—Zn1^i^	1.9335 (18)
			
O1—Zn1—O8^i^	126.97 (8)	C6—C5—H5	120.1
O1—Zn1—O5	113.18 (8)	C4—C5—H5	120.1
O8^i^—Zn1—O5	107.88 (9)	C9—O5—Zn1	118.61 (16)
O1—Zn1—O7^ii^	103.55 (8)	O7—C9—O5	121.7 (2)
O8^i^—Zn1—O7^ii^	98.21 (8)	O7—C9—C10	120.3 (2)
O5—Zn1—O7^ii^	103.04 (7)	O5—C9—C10	118.0 (2)
C1—O1—Zn1	128.96 (17)	O6—C10—C9	113.7 (2)
O2—C2—C1	115.7 (2)	O6—C10—H10A	108.8
O2—C2—H2A	108.4	C9—C10—H10A	108.8
C1—C2—H2A	108.4	O6—C10—H10B	108.8
O2—C2—H2B	108.4	C9—C10—H10B	108.8
C1—C2—H2B	108.4	H10A—C10—H10B	107.7
H2A—C2—H2B	107.4	C11—O6—C10	119.4 (2)
O8—C1—O1	126.4 (2)	O6—C11—C16	125.9 (2)
O8—C1—C2	113.8 (2)	O6—C11—C12	115.7 (2)
O1—C1—C2	119.8 (2)	C16—C11—C12	118.4 (3)
C3—O2—C2	117.3 (2)	C13—C12—C11	120.8 (3)
C3—C8—C7	121.6 (2)	C13—C12—Cl5	119.6 (2)
C3—C8—Cl2	119.6 (2)	C11—C12—Cl5	119.6 (2)
C7—C8—Cl2	118.8 (2)	C13—C14—C15	120.7 (3)
O2—C3—C8	116.8 (2)	C13—C14—Cl4	119.3 (2)
O2—C3—C4	124.7 (2)	C15—C14—Cl4	120.0 (3)
C8—C3—C4	118.5 (3)	C16—C15—C14	119.4 (3)
C8—C7—C6	118.2 (3)	C16—C15—H15	120.3
C8—C7—H7	120.9	C14—C15—H15	120.3
C6—C7—H7	120.9	C14—C13—C12	119.7 (3)
C3—C4—C5	120.6 (3)	C14—C13—H13	120.2
C3—C4—H4	119.7	C12—C13—H13	120.2
C5—C4—H4	119.7	C15—C16—C11	121.1 (3)
C5—C6—C7	121.3 (3)	C15—C16—H16	119.5
C5—C6—Cl3	119.7 (2)	C11—C16—H16	119.5
C7—C6—Cl3	119.0 (2)	C9—O7—Zn1^iii^	135.66 (16)
C6—C5—C4	119.8 (3)	C1—O8—Zn1^i^	144.34 (19)

Symmetry codes: (i) −*x*+1, −*y*+1, −*z*+1; (ii) *x*+1, *y*, *z*; (iii) *x*−1, *y*, *z*.

### Hydrogen-bond geometry (Å, °)

**Table:**

*D*—H···*A*	*D*—H	H···*A*	*D*···*A*	*D*—H···*A*
C2—H2B···Cl2^iv^	0.97	2.73	3.610 (3)	151

Symmetry codes: (iv) −*x*+1, −*y*+2, −*z*+1.

## References

[bb1] Bruker (2004). *APEX2 *and *SAINT* Bruker AXS Inc, Madison, Wisconsin, USA.

[bb2] Huang, F.-P., Yu, Q., Bian, H. D. & Yan, S.-P. (2008). *Polyhedron*, **27**, 3160–3166.

[bb3] Qin, J., Ma, J.-P., Liu, L.-L., Huang, R.-Q. & Dong, Y.-B. (2009). *Acta Cryst.* C**65**, m66–m68. 10.1107/S0108270109000341 19190373

[bb4] Reineke, T. M., Eddaoudi, M., O’Keeffe, M. & Yaghi, O. M. (1999). *Angew. Chem. Int. Ed.* **38**, 2590–2594. 10.1002/(sici)1521-3773(19990903)38:17<2590::aid-anie2590>3.0.co;2-h 10508349

[bb5] Sheldrick, G. M. (1996). *SADABS* University of Göttingen, Germany.

[bb6] Sheldrick, G. M. (2008). *Acta Cryst.* A**64**, 112–122. 10.1107/S0108767307043930 18156677

[bb7] Xiong, R.-G., Xue, X., Zhao, H., You, X.-Z., Abrahams, B. F. & Xue, Z. (2002). *Angew. Chem. Int. Ed.* **41**, 3800–3805. 10.1002/1521-3773(20021018)41:20<3800::AID-ANIE3800>3.0.CO;2-3 12386852

